# Supplementary material for: NOTCH Activation via gp130/STAT3 Signaling Confers Resistance to Chemoradiotherapy
Source: Cancers (Basel). 2021 Jan 26;13(3):455. doi: 10.3390/cancers13030455 (PMC7865718; doi:10.3390/cancers13030455)
Supplement: Supplementary file 1 [file cancers-13-00455-s001.pdf]

cancers

MDPI

Supplementary Materials

NOTCH activation via gp130/STAT3 signaling confers resistance to chemoradiotherapy

Kristin Koerdel, Melanie Spitzner, Thomas Meyer, Niklas Engels, Florian Krause, Jochen Gaedcke, Lena-Christin Conradi, Martin Haubrock, Tim Beißbarth, Andreas Leha, Steven A. Johnsen, B. Michael Ghadimi, Stefan Rose-John, Marian Grade and Jürgen Wienands

Supplementary Figures and Figure Legends

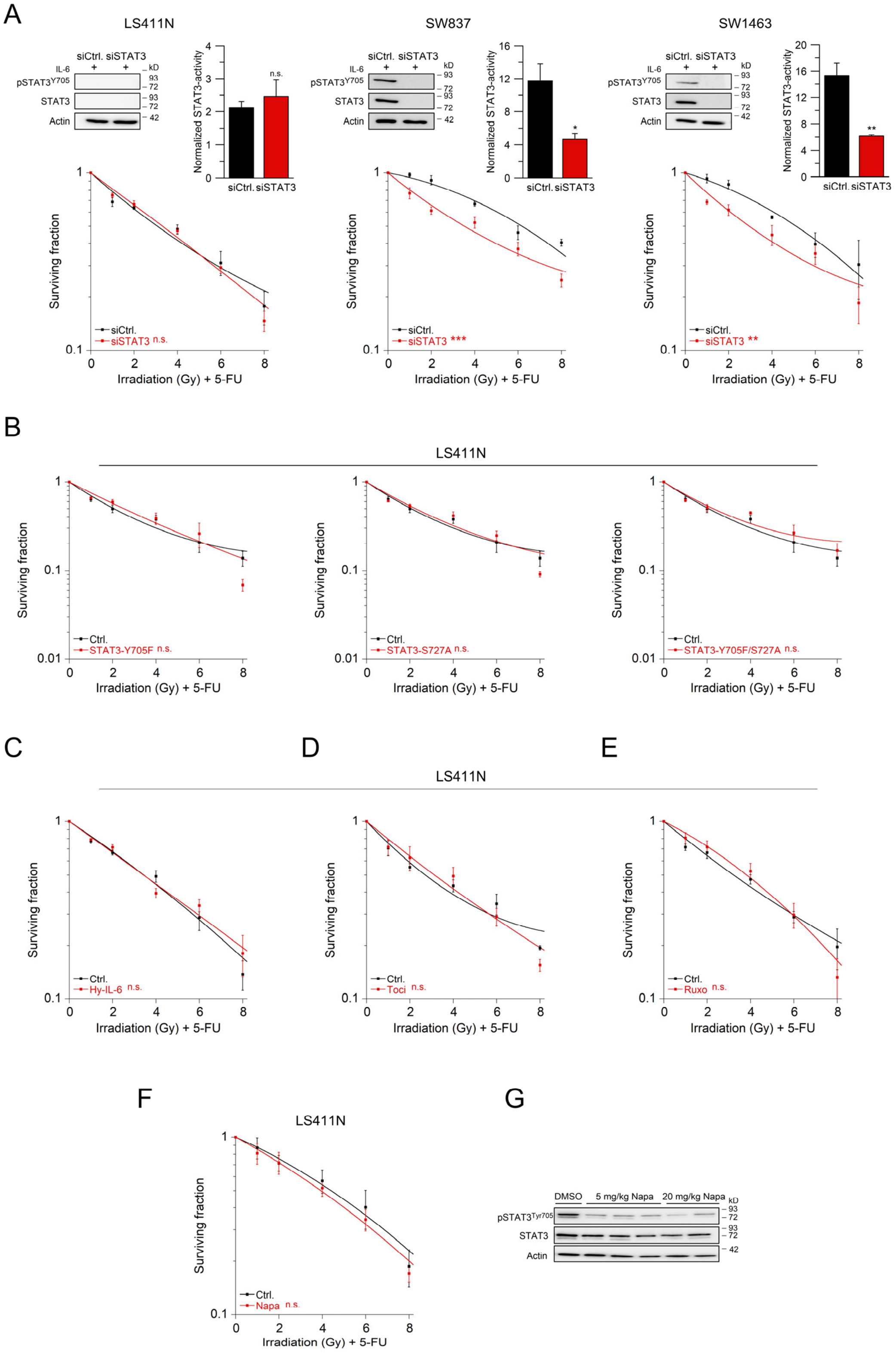

Supplementary Fig. S1. Transcriptionally active STAT3 is required for CRT resistance. (**A**) Indicated cell lines were treated with siRNA against STAT3 or control siRNA, and subsequently analyzed for inducible phosphorylation or expression of STAT3 by immunoblotting (upper left), or monitored for inducible STAT3 transcriptional activity (upper right), or were colony formation assay (CFA)-cultured to measure their survival following irradiation in the presence of 5-FU (lower graph). (**B**) STAT3-deficient LS411N cells were transfected with empty control vector (Ctrl.) or expression constructs encoding HA-tagged versions of STAT3 variants harboring the indicated amino acid exchanges. CFA survival of these transfectants after irradiation with the indicated doses (Gy) in the presence of 5-FU. (**C-E**) LS411N cells were left untreated or treated with (**C**) Hyper-IL-6 (Hy-IL-6), (**D**) tocilizumab (Toci), or (**E**) ruxolitinib (Ruxo), and analyzed for CFA survival after CRT. (**F**) LS411N cells were left untreated or treated with the STAT3 phosphorylation inhibitor napabucasin (Napa), and analyzed for their CFA survival after CRT. Data are presented as mean ± s.e.m., from at least n=3 independent biological replicates. * $p < 0.05$, ** $p < 0.01$, *** $p < 0.001$, unpaired two-sample Student's *t*-test or two-way analysis of variance (ANOVA). For *P*-values see Table S1. (**G**) To establish effective concentrations for in vivo experiments, xenotransplanted tumors of SW1463 were either treated with DMSO, or with two different concentrations of napabucasin: 5 mg/kg and 20 mg/kg, respectively. After three weeks of treatment, tumor-bearing mice were sacrificed 1 hour after oral application of either DMSO or napabucasin, and Western blot analysis was performed to confirm inhibition of STAT3 phosphorylation.

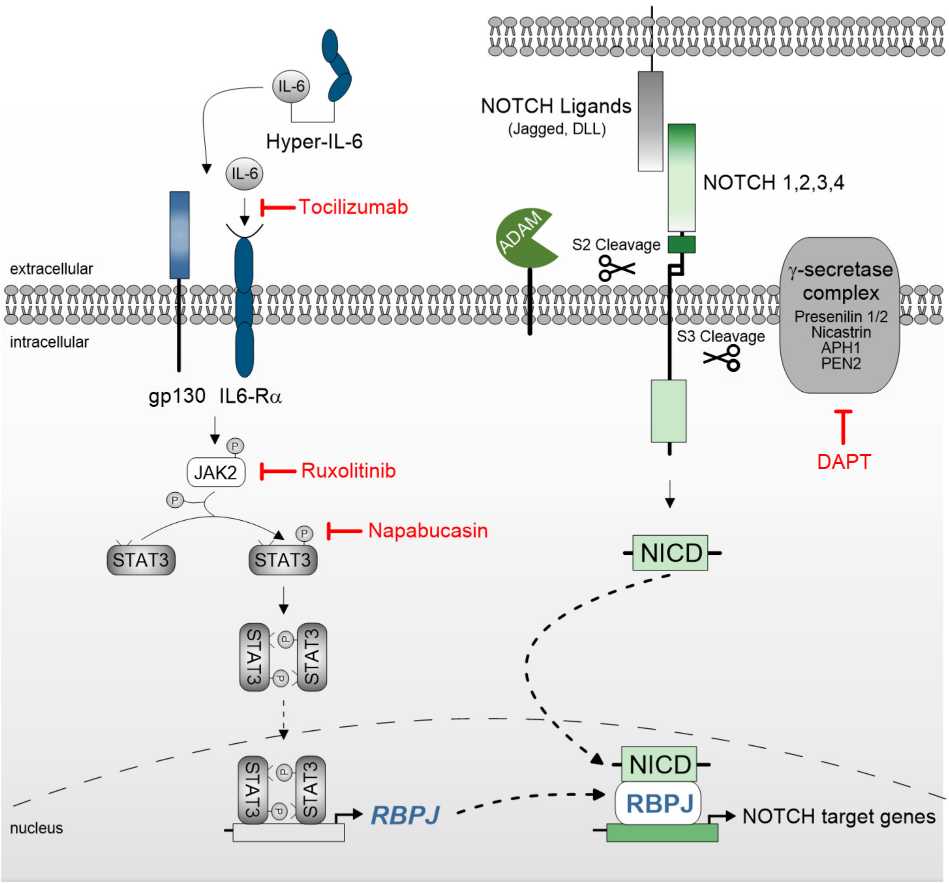

Supplementary Fig. S2. Inflammatory gp130/STAT3 signaling and the NOTCH/RBPJ pathway act in concert to block responsiveness to chemoradiotherapy. (left part) Ligated cytokine receptors of the gp130 family activate Janus tyrosine kinases (JAKs) to phosphorylate STAT3 that in turn dimerizes and translocates into the nucleus to regulate expression of STAT3 target genes by binding to specific docking sites called interferon-gamma activated sequence (GAS) [1-7]. Hyper-IL-6 represents a chimeric fusion protein encompassing IL-6 and the soluble IL-6 receptor chain [8]. (right part) Following ligation of NOTCH receptors on the cell surface by DELTA/Jagged ligands, NICD becomes proteolytically cleaved by ADAM family members (γ-secretase complex) and assembles with the transcription factor subunit RBPJ in the nucleus to drive expression of NOTCH target genes [9,10]. Pharmaceutical inhibitors used in this study are depicted in red.

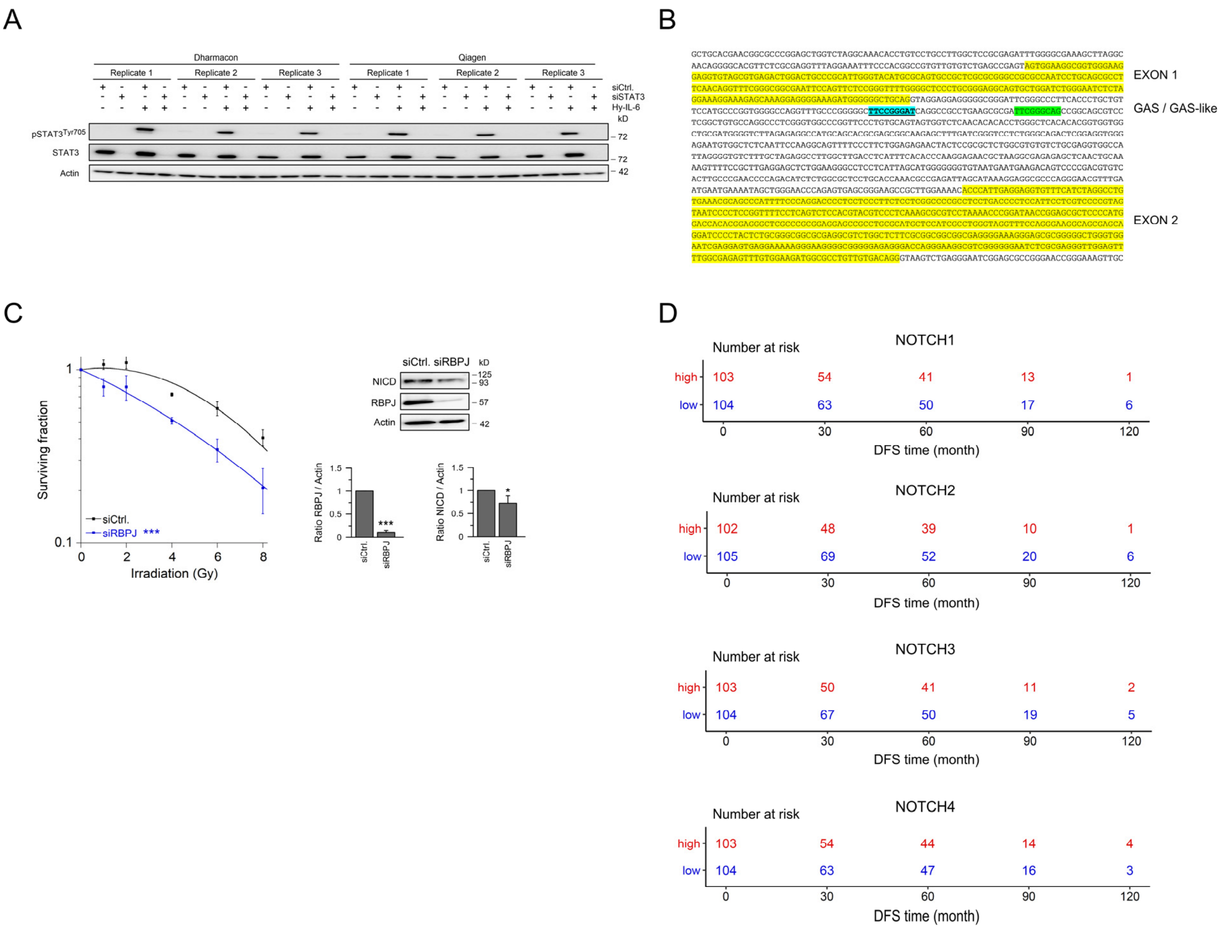

Supplementary Fig. S3. The Hyper-IL-6/STAT3 axis connects with the RBPJ/NOTCH pathway. (**A**) Immunoblot analysis of siRNA-treated SW837 cells with the indicated antibodies for three experimental conditions: (a) cellular stimulation with Hyper-IL-6 either in the presence of STAT3, or (b) upon siRNA-mediated STAT3 silencing, and (c) targeted STAT3 expression without further stimulation. (**B**) Cut out of the promotor region sequence of RBPJ. Exons 1 and 2 are highlighted in yellow, the gamma-interferon-activation sequence (GAS) in turquoise and a GAS-like element in green. (**C**) CFA of Hyper-IL-6-stimulated SW837 cells after RNAi-mediated silencing of RBPJ. Representative Western blot analysis with the indicated antibodies. Data are presented as mean ± s.e.m., from at least n=3 independent biological replicates. * $p < 0.05$, *** $p < 0.001$, unpaired two-sample Student's *t*-test or two-way analysis of variance (ANOVA). (**D**) Number of patients included for the correlation of gene expression (*NOTCH1-4*) with disease-free survival for each time point (months) and the two groups (high expression vs. low expression).

Figure 1A

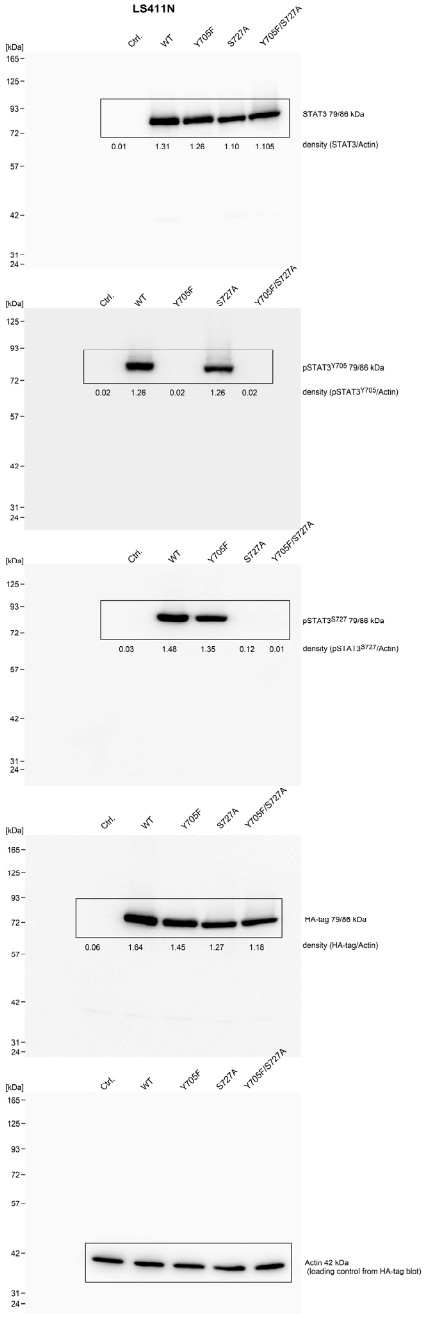

Figure 1B

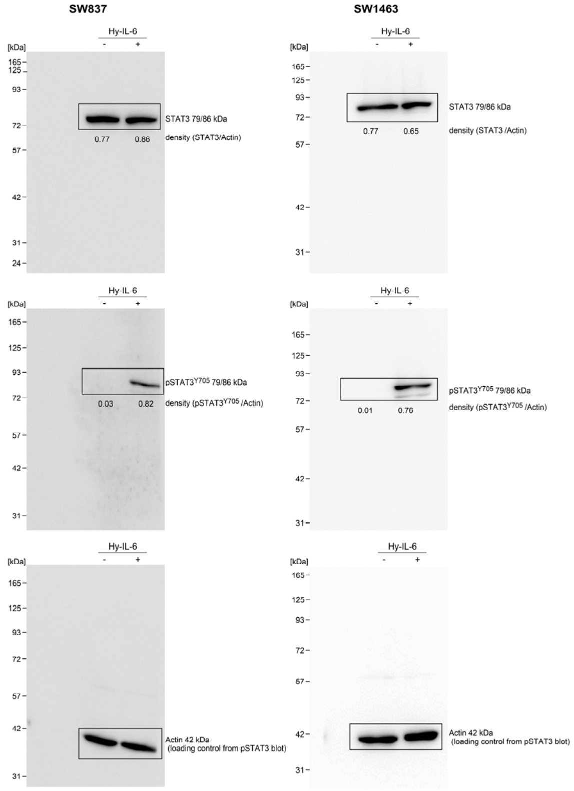

Figure 1C

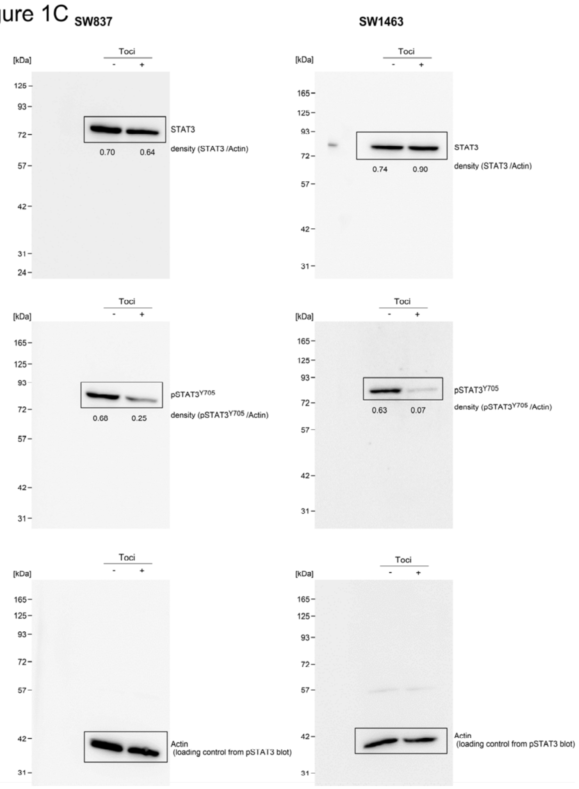

Figure 1D

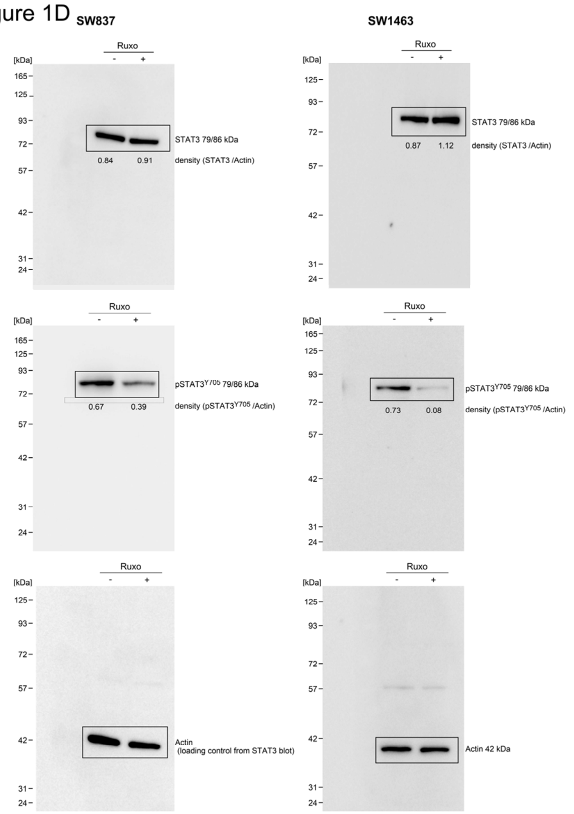

Figure 2A

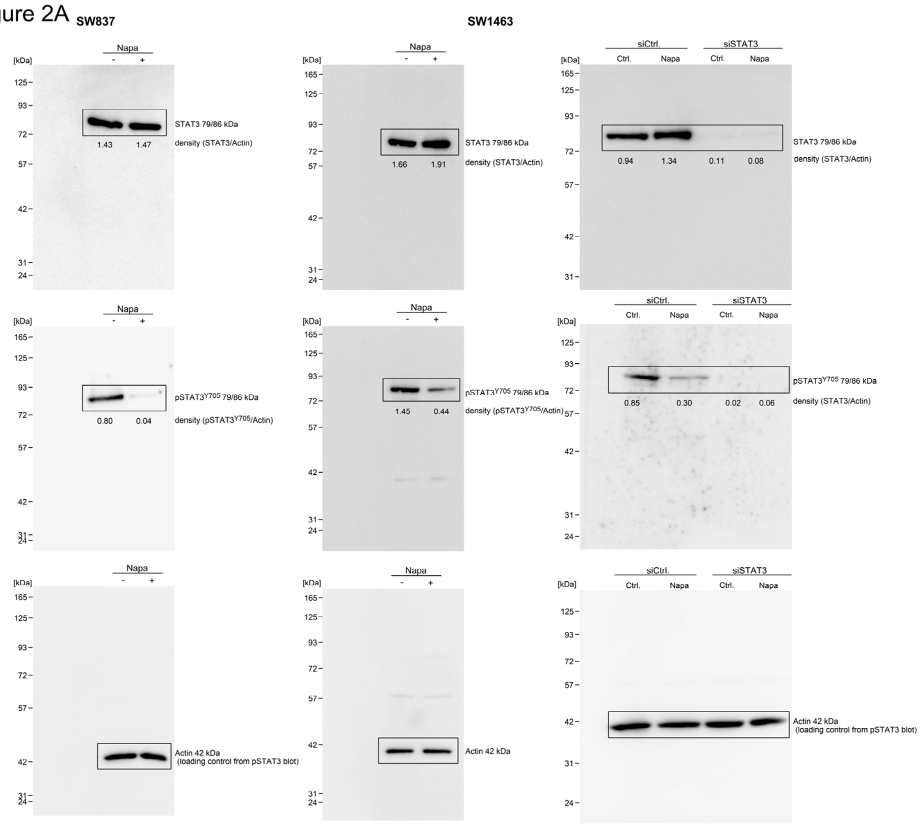

Figure 3A

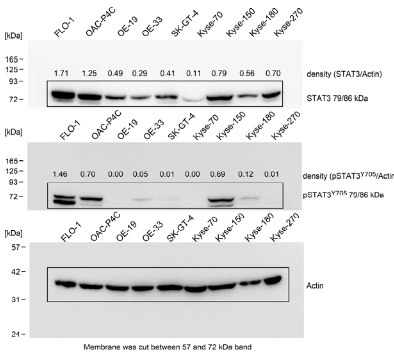

Figure 3B

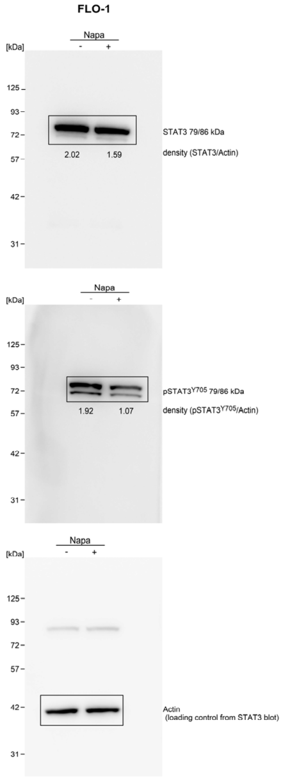

Figure 3C

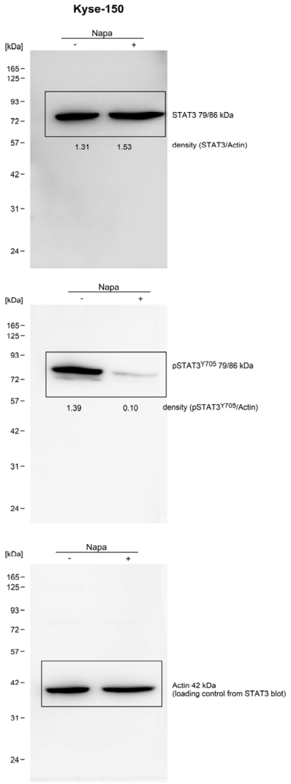

Figure 5C

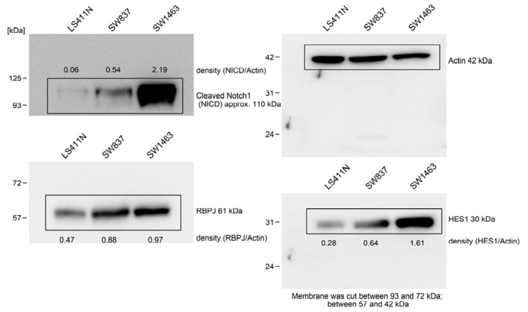

Figure 5D

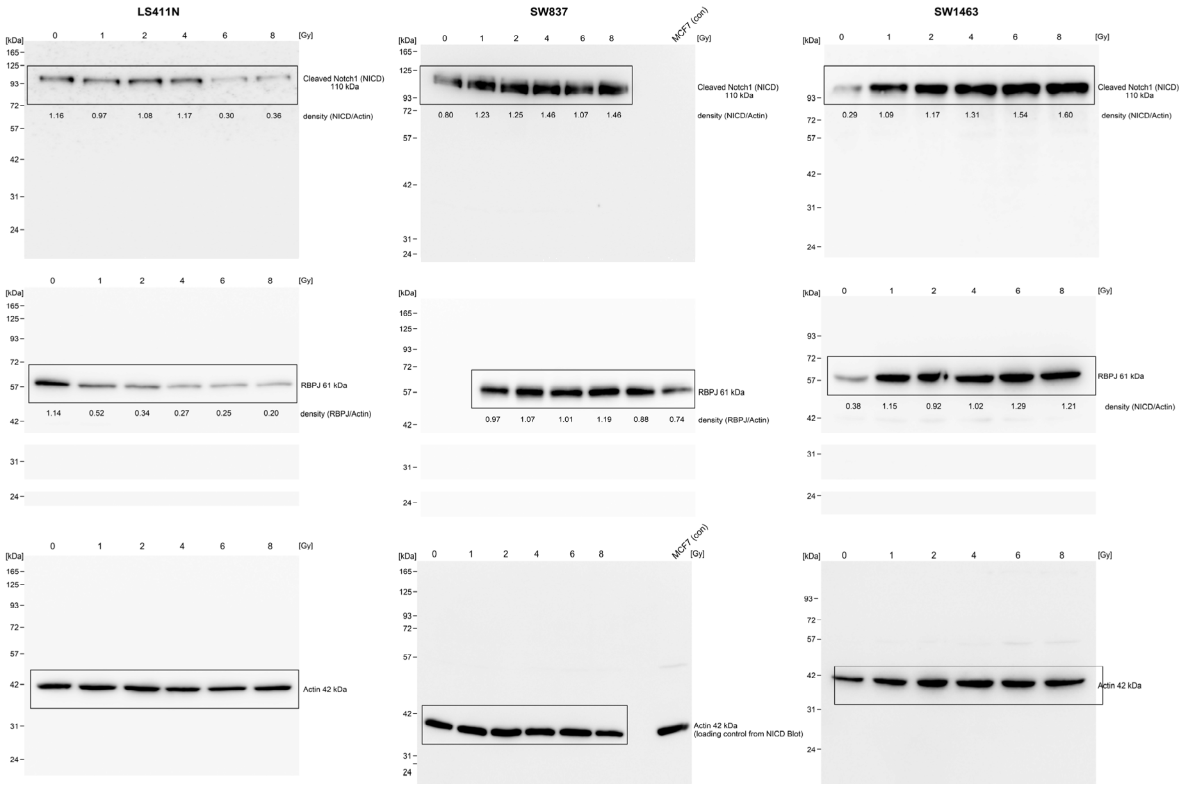

Figure 5E

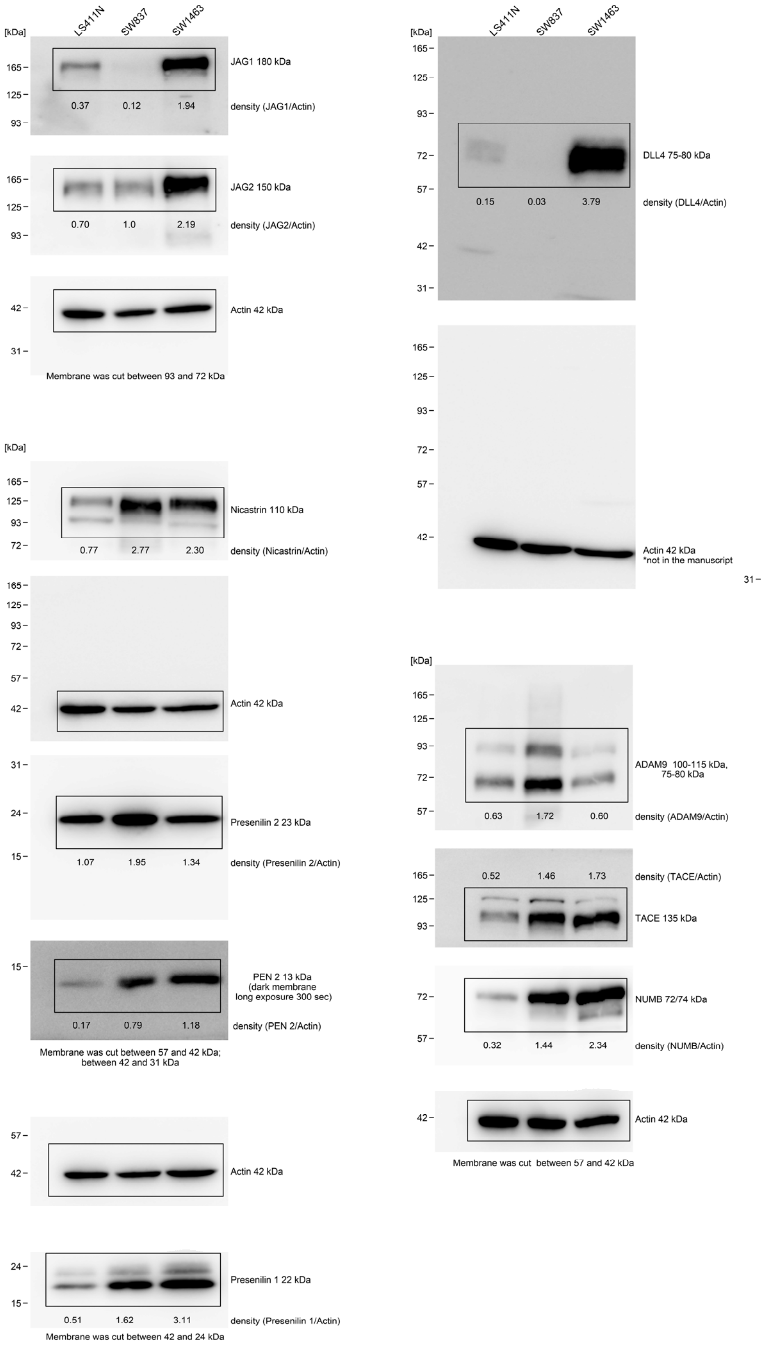

LS411N
SW837
SW1463
[kDa]
JAG1 180 kDa
0.37 0.12 1.94
density (JAG1/Actin)
JAG2 150 kDa
0.70 1.0 2.19
density (JAG2/Actin)
Actin 42 kDa
Membrane was cut between 93 and 72 kDa
Nicastrin 110 kDa
0.77 2.77 2.30
density (Nicastrin/Actin)
Actin 42 kDa
Presenilin 2 23 kDa
1.07 1.95 1.34
density (Presenilin 2/Actin)
PEN 2 13 kDa
(dark membrane
long exposure 300 sec)
0.17 0.79 1.18
density (PEN 2/Actin)
Membrane was cut between 57 and 42 kDa;
between 42 and 31 kDa
Actin 42 kDa
Presenilin 1 22 kDa
0.51 1.62 3.11
density (Presenilin 1/Actin)
Membrane was cut between 42 and 24 kDa
DLL4 75-80 kDa
0.15 0.03 3.79
density (DLL4/Actin)
Actin 42 kDa
*not in the manuscript
ADAM9 100-115 kDa,
75-80 kDa
0.63 1.72 0.60
density (ADAM9/Actin)
0.52 1.46 1.73
density (TACE/Actin)
TACE 135 kDa
NUMB 72/74 kDa
0.32 1.44 2.34
density (NUMB/Actin)
Actin 42 kDa
Membrane was cut between 57 and 42 kDa

Figure S1A

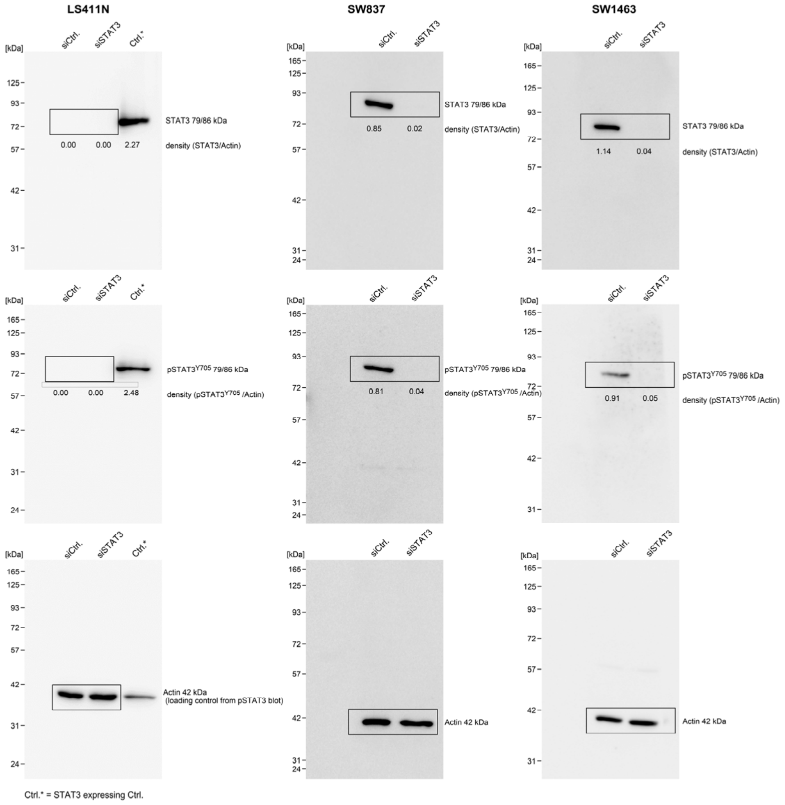

Figure S1G

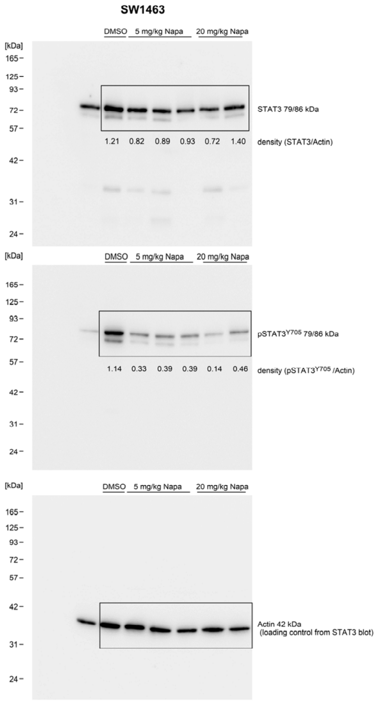

Figure S3A

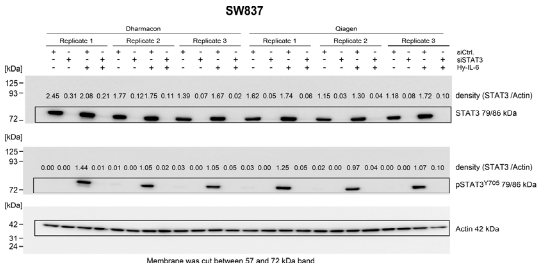

Figure S3C

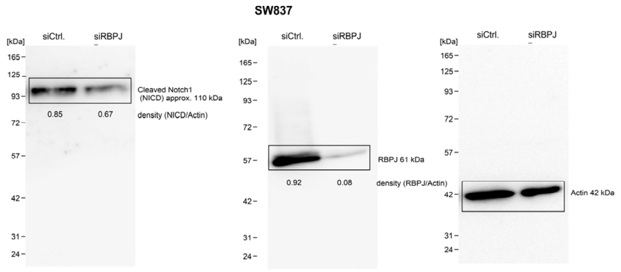

Supplementary Fig. S4. Original immunoblotting images presented in the paper.

Supplementary Tables

Supplementary Table S1. *P*-values of irradiation experiments and dual luciferase reporter assays (both in vitro), in vivo experiments, and patient survival data.

Cell line (*in vitro*)	Treatment	*P*-value CRT *	*P*-value DLR †
LS411N	siCtrl. vs. siSTAT3	0.9122 (Fig. S1A)	0.5696 (Fig. S1A)
SW837	siCtrl. vs. siSTAT3	2.032×10^{-8} (Fig. S1A)	0.0324 (Fig. S1A)
SW1463	siCtrl. vs. siSTAT3	0.0010 (Fig. S1A)	0.0082 (Fig. S1A)
LS411N	Ctrl. vs. STAT3-WT	0.0409 (Fig. 1A)	7.356×10^{-5} (Fig. 1A)
LS411N	Ctrl. vs. STAT3-Y705F	0.5651 (Fig. S1B)	0.0227 (Fig. 1A)
LS411N	Ctrl. vs. STAT3-S727A	0.3982 (Fig. S1B)	0.0026 (Fig. 1A)
LS411N	Ctrl. vs. STAT3-Y705F/S727A	0.1841 (Fig. S1B)	0.0091 (Fig. 1A)
LS411N	Ctrl. vs. Hy-IL-6	0.8245 (Fig. S1C)	n.a.
SW837	Ctrl. vs. Hy-IL-6	0.0216 (Fig. 1B)	0.0104 (Fig. 1B)
SW1463	Ctrl. vs. Hy-IL-6	0.0005 (Fig. 1B)	0.0096 (Fig. 1B)
LS411N	Ctrl. vs. Tocilizumab	0.9585 (Fig. S1D)	n.a.
SW837	Ctrl. vs. Tocilizumab	0.0323 (Fig. 1C)	0.0179 (Fig. 1C)
SW1463	Ctrl. vs. Tocilizumab	2.666×10^{-6} (Fig. 1C)	0.0207 (Fig. 1C)
LS411N	Ctrl. vs. Ruxolitinib	0.8581 (Fig. S1E)	n.a.
SW837	Ctrl. vs. Ruxolitinib	1.891×10^{-7} (Fig. 1D)	0.0093 (Fig. 1D)
SW1463	Ctrl. vs. Ruxolitinib	0.0001 (Fig. 1D)	0.0242 (Fig. 1D)
LS411N	Ctrl. vs. Napabucasin	0.5951 (Fig. S1F)	n.a.
SW837	Ctrl. vs. Napabucasin	0.0032 (Fig. 2A)	0.0307 (Fig. 2A)
SW1463	Ctrl. vs. Napabucasin	5.567×10^{-6} (Fig. 2A)	0.0044 (Fig. 2A)
SW1463	siCtrl. + DMSO vs. siSTAT3 + DMSO	4.111×10^{-5} (Fig. 2A)	n.a.
SW1463	siCtrl. + DMSO vs. siCtrl. + Napabucasin	2.608×10^{-11} (Fig. 2A)	n.a.
SW1463	siCtrl. + DMSO vs. siSTAT3 + Napabucasin	1.634×10^{-11} (Fig. 2A)	n.a.
FLO-1	Ctrl. vs. Napabucasin	1.189×10^{-6} (Fig. 3B)	n.a.
Kyse-150	Ctrl. vs. Napabucasin	1.774×10^{-10} (Fig. 3C)	n.a.
SW837	siCtrl. vs. siRBPJ	0.0041 (Fig. 5B)	n.a.
SW837	siCtrl. vs. siSTAT3	0.0026 (Fig. 5B)	n.a.
SW837	siCtrl. vs. siRBPJ + siSTAT3	0.0036 (Fig. 5B)	n.a.
SW837	siCtrl. + Hy-IL-6 vs. siRBPJ + Hy-IL-6‡	7.958×10^{-7} (Fig. S3C)	n.a.
SW837	siCtrl. + DMSO vs. siRBPJ + DMSO	0.0008 (Fig. 5F)	n.a.
SW837	siCtrl. + DMSO vs. siCtrl. + DAPT	0.0033 (Fig. 5F)	n.a.
SW837	siCtrl. + DMSO vs. siRBPJ + DAPT	0.0336 (Fig. 5F)	n.a.

Cell line (*in vivo*)	Treatment	*P*-value tumor volume	*P*-value tumor volume (end of treatment) †
SW1463	DMSO vs. Napabucasin (treatment period)	0.7899 (Fig. 2D) §	0.3575 (Fig. 2D)
SW1463	DMSO + CRT vs. Napabucasin + CRT (treatment period)	< 0.0001 (Fig. 2E) §	6.668×10^{-5} (Fig. 2E)
SW1463	DMSO + CRT vs. Napabucasin + CRT (post treatment)	< 0.0001 (Fig. 2F) §	n.a.
SW1463	DMSO + CRT vs. Napabucasin + CRT (post treatment)	0.013 (Fig. 2G) $	n.a.

Gene name	Patient group	*P*-value DFS &
NOTCH1	low vs. high mRNA expression	0.8980 (Fig. 5G)
NOTCH2	low vs. high mRNA expression	0.0036 (Fig. 5G)
NOTCH3	low vs. high mRNA expression	0.0395 (Fig. 5G)
NOTCH4	low vs. high mRNA expression	0.0122 (Fig. 5G)

* two-way analysis of variance (ANOVA), † unpaired two-sample Students *t*-test, ‡ only radiotherapy, § mixed-effects analysis using Tukey's multiple comparisons test, $ Log-rank (Mantel-Cox) test, & Cox proportional hazards model, CRT = chemoradiotherapy, DLR = dual luciferase reporter assay, WT = wild type, n.a. = not applicable; Hy-IL-6 = Hyper-IL-6, DSF = disease free survival

Supplementary Table S2. Pathway overrepresentation analysis identifies biologically annotated pathways using Gene Ontology terms.

	GO ID	Pathway description	Set size	NES	*P*-value
siCtrl. vs. siCtrl. + Hy-IL-6	GO:0010499	proteasomal ubiquitin-independent protein catabolic process	22	2.135	0.001
	GO:0046856	phosphatidylinositol dephosphorylation	21	2.013	0.001
	GO:0046839	phospholipid dephosphorylation	28	1.948	0.001
	GO:0071675	regulation of mononuclear cell migration	21	1.881	0.001
	GO:0048246	macrophage chemotaxis	18	1.880	0.001
	GO:0045649	regulation of macrophage differentiation	12	1.871	0.002
	GO:0019883	antigen processing and presentation of endogenous antigen	10	1.848	0.004
	GO:0001914	regulation of T cell mediated cytotoxicity	10	1.843	0.004
	GO:0070233	negative regulation of T cell apoptotic process	13	1.835	0.001
	GO:1903205	regulation of hydrogen peroxide-induced cell death	20	1.831	0.002
	GO:0060547	negative regulation of necrotic cell death	10	1.831	0.004
	GO:0007263	nitric oxide mediated signal transduction	11	1.830	0.004
	GO:0072676	lymphocyte migration	30	1.797	0.001
	GO:1905517	macrophage migration	19	1.776	0.002
	GO:0010907	positive regulation of glucose metabolic process	22	1.757	0.002
	GO:0071674	mononuclear cell migration	29	1.740	0.001
	GO:0046519	sphingoid metabolic process	12	1.738	0.006
	GO:1904816	positive regulation of protein localization to chromosome, telomeric region	11	1.737	0.007
	GO:0009396	folic acid-containing compound biosynthetic process	11	1.731	0.007
	GO:2000811	negative regulation of anoikis	11	1.722	0.007
siCtrl. vs. siSTAT3	GO:0006614	SRP-dependent cotranslational protein targeting to membrane	91	2.423	0.001
	GO:0031055	chromatin remodeling at centromere	36	2.403	0.001
	GO:0006613	cotranslational protein targeting to membrane	96	2.399	0.001
	GO:0070268	cornification	52	2.387	0.001
	GO:0034080	CENP-A containing nucleosome assembly	34	2.385	0.001
	GO:0061641	CENP-A containing chromatin organization	34	2.385	0.001
	GO:0072599	establishment of protein localization to endoplasmic reticulum	104	2.278	0.001
	GO:0045047	protein targeting to ER	101	2.264	0.001
	GO:1905214	regulation of RNA binding	11	2.231	0.001
	GO:0031424	keratinization	61	2.221	0.001
	GO:0048246	macrophage chemotaxis	18	2.179	0.001
	GO:0043486	histone exchange	44	2.177	0.001
	GO:0061844	antimicrobial humoral immune response mediated by antimicrobial peptide	18	2.170	0.001
	GO:0070098	chemokine-mediated signaling pathway	12	2.147	0.001
	GO:0010888	negative regulation of lipid storage	12	2.138	0.001
	GO:0000184	nuclear-transcribed mRNA catabolic process, nonsense-mediated decay	118	2.135	0.001
	GO:0036151	phosphatidylcholine acyl-chain remodeling	15	2.134	0.001
	GO:0034724	DNA replication-independent nucleosome organization	45	2.130	0.001
	GO:0022616	DNA strand elongation	26	2.127	0.001
	GO:0019730	antimicrobial humoral response	34	2.117	0.001

**siCtrl. + Hy-IL-6 vs. siSTAT3 + Hy-IL-6**	GO:0000045	autophagosome assembly	82	1.737	0.001
	GO:0000184	nuclear-transcribed mRNA catabolic process, nonsense-mediated decay	118	2.047	0.001
	GO:0000819	sister chromatid segregation	222	1.453	0.001
	GO:0000956	nuclear-transcribed mRNA catabolic process	201	1.550	0.001
	GO:0001666	response to hypoxia	248	1.394	0.001
	GO:0001816	cytokine production	416	1.481	0.001
	GO:0001817	regulation of cytokine production	380	1.391	0.001
	GO:0001959	regulation of cytokine-mediated signaling pathway	116	1.814	0.001
	GO:0002181	cytoplasmic translation	83	1.877	0.001
	GO:0002221	pattern recognition receptor signaling pathway	124	1.767	0.001
	GO:0002224	toll-like receptor signaling pathway	88	1.918	0.001
	GO:0002237	response to molecule of bacterial origin	184	1.501	0.001
	GO:0002250	adaptive immune response	166	1.521	0.001
	GO:0002253	activation of immune response	358	1.439	0.001
	GO:0002263	cell activation involved in immune response	456	1.456	0.001
	GO:0002274	myeloid leukocyte activation	421	1.450	0.001
	GO:0002275	myeloid cell activation involved in immune response	380	1.421	0.001
	GO:0002283	neutrophil activation involved in immune response	349	1.366	0.001
	GO:0002366	leukocyte activation involved in immune response	453	1.464	0.001
	GO:0002443	leukocyte mediated immunity	476	1.486	0.001

GO = Gene Ontology, NES = Normalized Enrichment Score, Hy-IL-6 = Hyper-IL-6

Supplementary Table S3. Opposite direction analysis (ODA). Fifty-five differentially regulated genes with an FDR < 0.05 were upregulated after pathway stimulation with Hy-IL-6 and, simultaneously but inversely, downregulated after STAT3 inhibition, and *vice versa*.

		**siCtrl. vs. siCtrl. + Hy-IL-6**		**siCtrl. vs. siSTAT3**		**siCtrl. + Hy-IL-6 vs. siSTAT3 + Hy-IL-6**	
**Gene name**	**Description**	**Log$_2$FC**	**FDR**	**Log$_2$FC**	**FDR**	**Log$_2$FC**	**FDR**
STAT3	signal transducer and activator of transcription 3<br>HGNC:11364	0.909	5.084x10^{-6}	-4.0756	2.374x10^{-17}	-4.5646	2.440x10^{-18}
CFI	complement factor I<br>HGNC:5394	3.774	3.845x10^{-8}	-1.2691	1.528x10^{-2}	-4.4856	2.125x10^{-9}
HTR3A	5-hydroxytryptamine receptor 3A<br>HGNC:5297	3.216	5.472x10^{-7}	-1.6959	2.093x10^{-3}	-4.1138	9.813x10^{-9}
SERPINB4	serpin family B member 4<br>HGNC:10570	2.009	7.913x10^{-6}	-1.5879	9.085x10^{-05}	-3.3529	2.945x10^{-9}
SERPINB3	serpin family B member 3<br>HGNC:10569	1.515	3.555x10^{-4}	-1.5750	4.073x10^{-05}	-2.9516	1.150x10^{-8}
PLAT	plasminogen activator tissue type<br>HGNC:9051	2.167	1.001x10^{-7}	-0.8584	1.411x10^{-3}	-2.5951	2.463x10^{-9}
DUOX2	dual oxidase 2<br>HGNC:13273	1.613	4.833x10^{-2}	-1.2895	3.212x10^{-2}	-2.3913	3.988x10^{-4}
FILIP1L	filamin A interacting protein 1 like<br>HGNC:24589	1.925	1.106x10^{-7}	-0.5763	1.533x10^{-2}	-2.3236	2.468x10^{-9}
SOCS3	suppressor of cytokine signaling 3<br>HGNC:19391	1.708	4.789x10^{-7}	-0.7972	1.186x10^{-3}	-2.3064	2.125x10^{-9}
S100A9	S100 calcium binding protein A9<br>HGNC:10499	1.817	1.443x10^{-4}	-0.7509	3.350x10^{-2}	-2.2056	2.187x10^{-6}
MUC1	mucin 1 cell surface associated<br>HGNC:7508	2.250	3.227x10^{-12}	-0.3565	1.590x10^{-2}	-2.1290	3.274x10^{-12}
HLA-DMB	major histocompatibility complex class II DM beta<br>HGNC:4935	1.646	8.534x10^{-8}	-0.4458	2.464x10^{-2}	-2.0454	1.100x10^{-9}
PLXDC1	plexin domain containing 1<br>HGNC:20945	1.241	3.184x10^{-4}	-0.6011	2.258x10^{-2}	-1.9279	1.735x10^{-7}
ST6GALNAC2	ST6 N-acetylgalactosaminide alpha-2.6-sialyltransferase 2<br>HGNC:10867	0.666	4.473x10^{-2}	-1.1506	5.167x10^{-05}	-1.6701	6.247x10^{-7}
HLA-DRA	major histocompatibility complex class II DR alpha<br>HGNC:4947	1.166	7.585x10^{-8}	-0.4809	9.180x10^{-4}	-1.5459	2.395x10^{-10}
CFB	complement factor B<br>HGNC:1037	1.089	2.232x10^{-5}	-0.7056	4.641x10^{-4}	-1.4810	7.029x10^{-8}
HLA-DRB6	major histocompatibility complex. class II. DR beta 6 (pseudogene)<br>HGNC:4954	0.913	4.265x10^{-3}	-0.7936	2.236x10^{-3}	-1.4580	3.765x10^{-6}
HLA-DPA1	major histocompatibility complex class II DP alpha 1<br>HGNC:4938	1.197	3.244x10^{-6}	-0.4335	1.634x10^{-2}	-1.4199	7.638x10^{-8}
HLA-DRB1	major histocompatibility complex class II DR beta 1<br>HGNC:4948	0.865	6.692x10^{-4}	-0.6425	1.270x10^{-3}	-1.3520	3.581x10^{-7}
CEACAM1	carcinoembryonic antigen related cell adhesion molecule 1<br>HGNC:1814	0.696	6.595x10^{-3}	-0.8666	9.147x10^{-05}	-1.3443	5.582x10^{-7}
CCDC69	coiled-coil domain containing 69<br>HGNC:24487	0.548	1.094x10^{-2}	-0.9258	5.937x10^{-06}	-1.3299	4.764x10^{-8}
TMEM176A	transmembrane protein 176A<br>HGNC:24930	0.777	4.265x10^{-3}	-0.5471	8.733x10^{-3}	-1.2319	3.765x10^{-6}
GNA15	G protein subunit alpha 15<br>HGNC:4383	0.531	1.473x10^{-2}	-0.7051	1.174x10^{-4}	-1.2286	1.367x10^{-7}
DPYD	dihydropyrimidine dehydrogenase<br>HGNC:3012	0.917	2.937x10^{-3}	-0.4815	3.711x10^{-2}	-1.2282	1.566x10^{-5}
RAB27A	RAB27A member RAS oncogene family<br>HGNC:9766	0.754	2.374x10^{-4}	-0.4625	3.097x10^{-3}	-1.1752	9.638x10^{-8}
SBNO2	strawberry notch homolog 2<br>HGNC:29158	0.840	2.234x10^{-6}	-0.3386	6.416x10^{-3}	-1.1705	3.314x10^{-9}
HLA-DRB5	major histocompatibility complex class II DR beta 5<br>HGNC:4953	0.866	3.708x10^{-3}	-0.5945	8.975x10^{-3}	-1.1525	2.196x10^{-5}

HLA-DMA	major histocompatibility complex class II DM alpha<br>HGNC:4934	0.837	5.164x10^{-4}	-0.3598	4.181x10^{-2}	-1.1464	1.541x10^{-6}
SERPINA1	serpin family A member 1<br>HGNC:8941	0.736	3.708x10^{-3}	-0.6139	1.855x10^{-3}	-1.1421	3.426x10^{-6}
LINC01503	long intergenic non-protein coding RNA 1503<br>HGNC:51184	0.649	6.159x10^{-3}	-0.4230	1.824x10^{-2}	-1.0783	3.242x10^{-6}
BCL6	B cell CLL/lymphoma 6<br>HGNC:1001	0.650	6.719x10^{-4}	-0.6685	6.085x10^{-05}	-1.0728	1.692x10^{-7}
TMEM176B	transmembrane protein 176B<br>HGNC:29596	0.616	3.708x10^{-3}	-0.5656	8.829x10^{-4}	-1.0392	1.339x10^{-6}
SYT12	synaptotagmin 12<br>HGNC:18381	0.615	2.037x10^{-2}	-0.4561	1.937x10^{-2}	-1.0158	1.992x10^{-5}
STRIP2	striatin interacting protein 2<br>HGNC:22209	0.570	4.564x10^{-2}	-0.6261	2.934x10^{-3}	-0.9942	4.300x10^{-5}
ARHGAP5-AS1	ARHGAP5 antisense RNA 1 (head to head)<br>HGNC:20279	0.491	2.708x10^{-2}	-0.5456	1.418x10^{-3}	-0.9212	6.960x10^{-6}
RBPJ	recombination signal binding protein for immunoglobulin kappa J region<br>HGNC:5724	0.333	1.782x10^{-2}	-0.5075	3.479x10^{-05}	-0.8156	7.638x10^{-8}
CASP10	caspase 10<br>HGNC:1500	0.433	2.291x10^{-2}	-0.3425	1.434x10^{-2}	-0.8074	5.525x10^{-6}
HRH1	histamine receptor H1<br>HGNC:5182	0.468	9.459x10^{-4}	-0.4805	7.978x10-^{05}	-0.8021	1.367x10^{-7}
SSFA2	sperm specific antigen 2<br>HGNC:11319	0.346	3.754x10^{-2}	-0.5292	9.478x10^{-05}	-0.7787	1.339x10^{-6}
ELF3	E74 like ETS transcription factor 3<br>HGNC:3318	0.487	2.752x10^{-2}	-0.3778	1.819x10^{-2}	-0.7683	5.358x10^{-5}
GSDMB	gasdermin B<br>HGNC:23690	0.465	1.723x10^{-2}	-0.3194	2.500x10^{-2}	-0.7500	1.863x10^{-5}
IL18	interleukin 18<br>HGNC:5986	0.397	1.723x10^{-2}	-0.3125	1.106x10^{-2}	-0.6921	7.143x10^{-6}
MAP3K6	mitogen-activated protein kinase kinase kinase 6<br>HGNC:6858	0.373	4.050x10^{-3}	-0.4267	1.068x10^{-4}	-0.6896	3.743x10^{-7}
UBA7	ubiquitin like modifier activating enzyme 7<br>HGNC:12471	0.445	4.041x10^{-2}	-0.4368	5.727x10^{-3}	-0.6804	1.370x10^{-4}
TRIB2	tribbles pseudokinase 2<br>HGNC:30809	0.294	4.701x10^{-2}	-0.3671	9.808x10^{-4}	-0.6802	1.450x10^{-6}
NAMPT	nicotinamide phosphoribosyltransferase<br>HGNC:30092	0.352	2.177x10^{-2}	-0.2591	2.010x10^{-2}	-0.6445	5.814x10^{-6}
LDHA	lactate dehydrogenase A<br>HGNC:6535	0.367	1.162x10^{-2}	-0.2708	1.218x10^{-2}	-0.6128	6.661x10^{-6}
HK1	hexokinase 1<br>HGNC:4922	0.338	3.215x10^{-2}	-0.3396	3.611x10^{-3}	-0.5914	2.026x10^{-5}
CASP7	caspase 7<br>HGNC:1508	0.302	1.723x10^{-2}	-0.2995	1.953x10^{-3}	-0.5867	1.898x10^{-6}
IL4R	interleukin 4 receptor<br>HGNC:6015	0.244	4.988x10^{-2}	-0.3665	1.829x10^{-4}	-0.5081	5.521x10^{-6}
HIF1A	hypoxia inducible factor 1 subunit alpha<br>HGNC:4910	0.279	1.723x10^{-2}	-0.2189	1.120x10^{-2}	-0.4978	5.521x10^{-6}
PITPNM2	phosphatidylinositol transfer protein membrane associated 2<br>HGNC:21044	0.437	7.925x10^{-4}	-0.2476	1.124x10^{-2}	-0.4686	4.818x10^{-5}
TSTA3	tissue specific transplantation antigen P35B<br>HGNC:12390	0.382	5.754x10^{-3}	-0.2340	2.305x10^{-2}	-0.4356	2.027x10^{-4}
UAP1L1	UDP-N-acetylglucosamine pyrophosphorylase 1 like 1<br>HGNC:28082	-0.277	2.752x10^{-2}	0.3622	2.891x10^{-4}	0.5327	5.018x10^{-6}
GNAL	G protein subunit alpha L<br>HGNC:4388	-0.690	2.752x10^{-2}	0.8846	2.590x10^{-4}	1.1498	2.427x10^{-5}

ODA = opposite direction analysis, FDR = false discovery rate, Hy-IL-6 = Hyper-IL-6, FC = fold change

Supplementary Table S4. Sequences for electrophoretic mobility shift assay

Probe	Probe sequence	Size (bp)	Company
RBPJ (native)	Forward: CGGGGGCTTCCGGGATCAGGCC	22	Sigma-Aldrich
	Reverse: GGCCTGATCCCGGAAGCCCCCG	22	
RBPJ (mutated)	Forward: CGGGGGCCCTTGGTAGCAGGCC	22	Sigma-Aldrich
	Reverse: GGCCTGCTACCAAGGGCCCCCG	22	
M67	Forward: CGACATTTCCCGTAAATCTG	20	Sigma-Aldrich
	Reverse: CAGATTTACGGGAAATGTCG	20	

bp = base pair, red = mutation site

Supplementary Table S5. Clinical characteristics of rectal cancer patients.

Characteristics	Cohort (n = 207)
Age	
Years, median (range)	63 (36 – 82)
Sex	
Male, *n* (%)	144 (69.6)
Female, *n* (%)	63 (30.4)
DFS	
Follow-up time, month, median (range)	37 (0 – 188)
Reported events, *n* (%)	52 (25.1)
UICC Staging (2010)	
ypUICC 0, *n* (%)	35 (16.9)
ypUICC I, *n* (%)	56 (27.1)
ypUICC II, *n* (%)	48 (23.3)
ypUICC III, *n* (%)	49 (23.7)
ypUICC IV, *n* (%)	19 (9.2)

DSF = disease-free survival, UICC = Union International Contre le Cancer, ypUICC refers to histopathologic assessment of the resected specimens after completion of preoperative chemoradiotherapy.

Supplementary Table S6. ***NOTCH1-4*** **gene expression in pretherapeutic biopsies of 207 rectal cancer patients.**

Patient	*NOTCH1*	*NOTCH2*	*NOTCH3*	*NOTCH4*
Pat-001	11.04983	12.42652	14.05618	10.12461
Pat-002	9.731679	11.77624	10.68972	8.464393
Pat-003	9.866303	11.44283	11.17424	8.664112
Pat-004	10.619	11.62806	11.59862	8.888907
Pat-005	10.06748	11.78198	12.62927	8.980322
Pat-006	9.512866	11.56179	11.09134	9.5233
Pat-007	10.57264	9.982374	10.32905	8.873815
Pat-008	9.741313	11.83664	12.2693	9.082583
Pat-009	10.65923	11.39058	11.45918	9.030163
Pat-010	9.931382	11.54004	11.52335	9.040775
Pat-011	10.75885	11.78992	9.198518	7.894697
Pat-012	10.01728	12.65875	10.34408	8.496484
Pat-013	10.13775	12.46177	14.62135	10.70099
Pat-014	11.59681	11.34077	10.31917	7.994789
Pat-015	10.02977	11.52153	10.20449	8.998863
Pat-016	9.978194	11.39555	11.46496	8.997476
Pat-017	11.49236	12.38601	14.03451	9.462063
Pat-018	10.8295	11.8466	13.03207	9.994209
Pat-019	9.6303	11.60004	10.83162	9.0237
Pat-020	10.30427	11.90017	12.17044	9.175658
Pat-021	10.45717	11.64137	11.58937	8.88562
Pat-022	10.60579	11.87089	11.18083	8.131042
Pat-023	10.78997	11.88292	12.52527	9.620656
Pat-024	10.49149	11.66542	11.46714	9.296407
Pat-025	10.23944	11.80421	11.92088	9.592988
Pat-026	9.790014	12.28877	12.81091	9.861815
Pat-027	9.546565	12.14192	12.77906	9.859477
Pat-028	10.3077	11.43776	11.8797	8.395192
Pat-029	10.4904	11.84357	12.03801	9.501007
Pat-030	10.10474	11.736	12.04737	9.019728
Pat-031	10.52994	11.46	11.84619	9.279854
Pat-032	9.791291	11.70618	13.19024	9.868134
Pat-033	10.0853	12.41331	12.50665	9.299119
Pat-034	11.11913	11.85592	11.29051	8.922393
Pat-035	10.30887	11.50358	10.54498	8.101961
Pat-036	10.46729	11.34077	11.21751	8.427457
Pat-037	10.54829	11.63986	11.60112	9.032264
Pat-038	9.464504	11.64637	12.36324	8.816596
Pat-039	10.25306	12.25794	12.47007	9.191856
Pat-40	10.43973	11.25894	10.83935	8.295055
Pat-41	9.441094	11.56749	12.25179	9.307712
Pat-42	9.782012	11.67148	11.7108	9.165679
Pat-43	9.940676	11.41489	12.36161	9.462159
Pat-44	9.32709	11.64174	10.66274	8.203706
Pat-45	10.78582	11.4636	11.47179	9.186141
Pat-46	10.5176	11.40694	12.45749	9.228259
Pat-47	10.70882	11.51796	9.983373	8.889697
Pat-48	9.990982	11.67774	12.69773	9.220533
Pat-49	9.792462	12.426	14.48093	11.08546
Pat-050	10.2336	11.75141	12.21994	8.619593
Pat-051	10.40173	11.33854	10.85524	8.353989
Pat-052	9.360928	10.57228	10.62005	9.366539
Pat-053	10.73274	11.00727	11.84763	9.050194
Pat-054	10.50872	11.91642	12.99222	9.834343
Pat-055	10.48893	12.14215	11.79483	8.667029
Pat-056	9.914022	11.69362	11.71556	9.18874
Pat-057	10.01941	11.63701	12.3748	9.682885
Pat-058	9.311273	12.21572	13.14887	9.440572
Pat-059	9.342314	11.62089	11.10758	8.586617
Pat-060	10.03509	11.1908	11.4201	9.580409
Pat-061	9.602898	11.25725	10.20483	8.97369
Pat-062	10.58654	11.59445	11.63556	9.926953

Pat-063	10.22266	11.17094	10.57088	8.909016
Pat-064	10.47859	11.93545	12.3806	9.202566
Pat-065	10.17004	12.25691	12.21149	9.201538
Pat-066	10.0785	11.71538	9.953081	8.228883
Pat-067	9.732106	11.42327	10.84693	8.889216
Pat-068	10.3184	11.32069	11.24085	9.140126
Pat-069	9.214043	12.25431	12.2938	9.299507
Pat-070	9.704544	11.61319	11.53697	9.147175
Pat-071	10.30852	11.22388	12.29532	9.286154
Pat-072	10.13355	11.36414	10.3455	8.388921
Pat-073	9.753751	12.18803	12.50836	9.540645
Pat-074	10.39456	12.10307	13.13949	9.575417
Pat-075	10.02808	11.80004	11.68624	9.135354
Pat-076	10.50215	11.34315	10.83122	8.830078
Pat-077	10.15511	11.5122	12.72396	10.06895
Pat-078	10.02194	11.58251	11.38766	9.180558
Pat-079	9.460633	12.16179	12.32188	8.830078
Pat-080	10.73689	10.5853	10.62735	8.642144
Pat-081	10.96248	10.91182	9.393066	7.608275
Pat-082	9.671261	11.71849	12.55825	9.068364
Pat-083	11.0161	11.09479	11.38541	8.693859
Pat-084	10.469	11.14055	11.96355	9.619548
Pat-085	9.378398	11.7861	10.67318	8.466704
Pat-086	10.03009	11.34685	10.62018	8.103141
Pat-087	10.59062	11.83068	11.52737	9.208135
Pat-088	10.24116	11.80872	12.49072	9.114175
Pat-089	10.34958	11.35143	12.01944	9.232391
Pat-090	10.75184	11.62216	11.27176	8.975611
Pat-091	9.629353	11.90261	12.25843	9.466802
Pat-092	10.94048	11.54898	11.38135	9.390689
Pat-093	9.302066	11.80285	11.98355	9.035357
Pat-094	10.51146	11.78431	11.51185	9.415396
Pat-095	9.363129	12.44985	13.22625	9.630928
Pat-096	9.89424	11.23004	11.5786	8.992508
Pat-097	9.25675	12.1634	13.40567	9.537967
Pat-098	9.536062	11.54212	10.90167	8.773628
Pat-099	9.76409	11.33107	11.48038	9.039082
Pat-100	10.30639	12.46565	12.47965	9.246143
Pat-101	9.941321	10.86513	10.69198	8.223497
Pat-102	10.87343	12.2693	11.82064	8.628491
Pat-103	10.26182	11.63701	11.08403	8.589154
Pat-104	10.71797	11.96333	12.30615	8.866599
Pat-105	9.754769	11.60795	11.9383	9.114276
Pat-106	11.1558	11.95802	11.33697	8.577728
Pat-107	10.04605	11.73983	11.99583	9.479999
Pat-108	10.64443	11.29932	12.11101	8.755175
Pat-109	9.742656	11.72838	11.52705	9.569033
Pat-110	9.915313	11.80714	9.996324	8.897482
Pat-111	9.526179	12.32752	11.81303	9.064551
Pat-112	10.64022	10.56902	10.4619	7.879649
Pat-113	10.79648	12.13572	13.37394	9.863671
Pat-114	10.0973	12.13801	13.68009	10.33822
Pat-115	10.27148	11.67756	12.08386	9.513995
Pat-116	10.16494	11.72267	11.83008	8.807729
Pat-117	10.46751	11.39673	10.20699	8.432936
Pat-118	9.693494	11.96096	12.25967	9.723943
Pat-119	10.90415	11.82801	13.39346	9.842554
Pat-120	9.537352	11.48418	11.31376	8.730209
Pat-121	9.856208	12.41957	10.7387	6.884501
Pat-122	10.29003	12.36324	11.8958	9.130786
Pat-123	9.910039	11.1111	8.975137	8.365973
Pat-124	10.11935	11.24771	11.76574	9.678845
Pat-125	10.57704	11.30806	10.52725	8.561984
Pat-126	10.69198	10.62092	10.74184	8.210136
Pat-127	10.08584	11.72343	11.40773	9.21988
Pat-128	9.640998	12.42543	13.37394	10.9654
Pat-129	9.560869	11.82822	11.33139	9.029402
Pat-130	10.27901	12.55855	14.04023	10.16033

Pat-131	9.855954	12.06768	12.5995	9.954905
Pat-132	9.785206	12.02266	12.71394	9.105783
Pat-133	10.2007	11.44631	12.17235	8.952168
Pat-134	10.17317	11.65684	12.02834	8.957172
Pat-135	11.76532	11.25653	11.6785	8.764462
Pat-136	9.961864	11.65595	12.71916	9.495241
Pat-137	10.82563	11.67832	11.30572	9.22639
Pat-138	10.56324	10.91126	11.6785	8.588442
Pat-139	10.08851	11.85349	11.78491	9.152983
Pat-140	10.29728	11.4014	11.42545	8.53772
Pat-141	9.636398	11.61245	11.65781	9.107418
Pat-142	9.923858	12.02199	11.67812	8.756212
Pat-143	10.51933	11.67168	10.43265	8.099439
Pat-144	10.11696	12.69259	13.27998	10.1801
Pat-145	11.11008	10.80435	11.24235	8.346613
Pat-146	10.21924	11.16326	12.1926	9.293535
Pat-147	10.42602	10.60714	10.14505	7.461013
Pat-148	9.97115	12.36677	12.42356	9.475759
Pat-149	10.41184	11.45886	10.92035	8.873815
Pat-150	9.973145	11.16537	10.92006	7.415855
Pat-151	10.81367	11.24299	10.94606	8.456112
Pat-152	10.8931	11.26089	12.48407	8.957434
Pat-153	10.09105	10.61166	10.53661	9.391897
Pat-154	9.568923	13.00409	13.05767	9.321791
Pat-155	10.76208	11.60184	13.18555	9.581423
Pat-156	10.49077	12.93195	14.06182	10.49446
Pat-157	10.87839	10.88563	10.99438	8.729738
Pat-158	10.85445	11.19066	12.70108	9.98466
Pat-159	10.21882	12.0244	13.04971	9.957172
Pat-160	10.66106	11.24667	11.26368	9.034898
Pat-161	10.17443	11.32452	11.57417	9.236923
Pat-162	11.41828	10.82363	9.130384	7.839059
Pat-163	10.50922	10.48355	10.57486	7.878572
Pat-164	10.58477	11.27987	12.58363	9.05503
Pat-165	9.974461	12.12862	13.39582	10.18406
Pat-166	10.97595	11.17497	11.83685	8.506993
Pat-167	10.46876	11.37242	11.06653	8.640772
Pat-168	10.81224	11.86733	11.82861	9.142732
Pat-169	10.83419	11.60777	11.67014	8.20841
Pat-170	9.9182	11.87775	12.30823	8.857315
Pat-171	10.98292	12.04424	12.87089	9.189733
Pat-172	10.59173	11.23447	12.1256	9.449225
Pat-173	10.22872	11.76767	11.08258	8.941997
Pat-174	10.79369	10.94036	10.68484	8.34375
Pat-175	9.799353	11.56697	11.23557	8.097647
Pat-176	9.564991	12.18257	13.00791	10.23805
Pat-177	10.80944	11.50573	12.28248	9.082162
Pat-178	8.914651	11.58234	13.64526	10.49137
Pat-179	9.84086	11.74561	11.22794	8.863543
Pat-180	9.923089	11.46309	12.07893	9.134652
Pat-181	9.980772	10.99653	11.59024	9.311886
Pat-182	11.09739	11.95284	13.2375	9.967198
Pat-183	10.09607	11.54298	11.94585	9.427923
Pat-184	10.15055	11.39248	11.3376	9.249525
Pat-185	9.716082	11.41796	11.25223	8.977081
Pat-186	8.637104	11.20417	7.615348	7.118193
Pat-187	10.45817	11.80481	12.3311	10.04784
Pat-188	9.532492	12.25639	12.6581	10.60276
Pat-189	11.2728	11.30397	11.9342	9.375231
Pat-190	10.85658	10.73213	10.72371	8.63163
Pat-191	10.65615	12.29809	11.63196	8.58885
Pat-192	10.16614	11.23756	10.45129	9.201538
Pat-193	9.980772	11.36464	11.05905	9.584938
Pat-194	10.06134	11.40869	10.96055	8.451922
Pat-195	11.11536	11.58674	12.42759	9.311992
Pat-196	9.801015	11.97496	12.93933	9.580613
Pat-197	10.38528	11.31453	10.822	8.668519
Pat-198	9.935797	11.8906	12.18257	9.448909

Pat-199	10.27172	12.07213	12.25079	8.82771
Pat-200	10.96286	12.14076	14.16664	10.59162
Pat-201	10.28761	12.67851	12.76641	9.588122
Pat-202	9.528575	11.34747	13.73514	10.12363
Pat-203	10.58951	11.49372	9.402161	7.840662
Pat-204	10.09761	11.60433	11.65817	8.886712
Pat-205	10.23587	11.66427	12.78612	9.773912
Pat-206	11.66067	10.61581	8.945718	7.912341
Pat-207	10.64198	11.71813	12.76641	9.584479

Supplementary Materials and Methods

RNA interference

Transfections with siRNA were performed as described [11,12] with the following siRNA pools: negative control siRNA 1 (ON-TARGETplus Non-targeting Pool, Dharmacon Horizon Discovery, Cambridge, UK), or negative control siRNA 2 (AllStars Negative Control siRNA, Qiagen, Hilden, Germany), siSTAT3 or siRBPJ (ON-TARGETplus STAT3 siRNA, ON-TARGETplus RBPJ siRNA, Dharmacon).

siRNA sequences

Gene	Target Sequence	Size (bp)	Accession Number	Company	Catalogue number
Negative control (ON-TARGETplus)	UGGUUUACAUGUCGACUAA	19	n.a.	Dharmacon	D-001810-10
	UGGUUUACAUGUUGUGUGA	19	n.a.	Dharmacon	D-001810-10
	UGGUUUACAUGUUUUCUGA	19	n.a.	Dharmacon	D-001810-10
	UGGUUUACAUGUUUUCCUA	19	n.a.	Dharmacon	D-001810-10
STAT3 (Pool)	GAGAUUGACCAGCAGUAUA	19	NM_003150	Dharmacon	L-003544-00
	CAACAUGUCAUUUGCUGAA	19	NM_003150	Dharmacon	L-003544-00
	CCAACAAUCCCAAGAAUGU	19	NM_003150	Dharmacon	L-003544-00
	CAACAGAUUGCCUGCAUUG	19	NM_003150	Dharmacon	L-003544-00
RBPJ (Pool)	GUAGAGAGCCUUCAGUUGA	19	NM_203283	Dharmacon	L-007772-00
	CUCCCAAGAUUGAUAAUUA	19	NM_203283	Dharmacon	L-007772-00
	CCAGAUACUUGCAUGUAGA	19	NM_203283	Dharmacon	L-007772-00
	GGUCCGAAAUGAUGGAAUC	19	NM_203283	Dharmacon	L-007772-00
Negative control (AllStarsNEG)	CAGGGTATCGACGATTACAAA	21	n.a.	Qiagen	SI03650318
STAT3 (#7) *	CAGCCTCTCTGCAGAATTCAA	21	NM_003150	Qiagen	SI02662338
STAT3 (#8) *	CAGGCTGGTAATTTATATAAT	21	NM_003150	Qiagen	SI02662898

* siRNA STAT3 (#7) and siSTAT3 (#8) were pooled for RNA-Seq experiments, bp = base pair, n.a. = not applicable

Western blot analysis

Immunoblotting was performed as described [11]. For detection of phosphorylated STAT3, CRC cells were stimulated with either recombinant IL-6 (Biochrom, Germany) or Hyper-IL-6 (Hy-IL-6), esophageal cancer cells were left unstimulated, and lysed in Nonidet P-40 (NP-40) buffer. Following Bradford quantification, proteins were separated on 10% Bis-Tris PA gels and transferred to PVDF membrane (GE Healthcare, Little Chalfont, UK). Bound antibodies were detected with ImageQuant LAS 4000 mini CCD camera system (GE Healthcare). ImageJ software (version 1.52a, National Institutes of Health, USA) was used for quantification of protein band intensities. Original blot images and calculated band intensities are provided in Supplementary Figure S4.

Antibodies for Western blot analysis

Protein	clone	Host	Size (kD)	Dilution	Incubation time	Company	Catalogue number
Actin	Polyclonal	Rabbit	42	1 : 10,000	Over night	Sigma-Aldrich	A2066
pSTAT3^{Tyr705}	D3A7	Rabbit	79/86	1 : 1,500	Over night	Cell Signaling	9145
pSTAT3^{Ser727}	Polyclonal	Rabbit	79/86	1 : 1,000	Over night	Cell Signaling	9134
STAT3	D3Z2G	Rabbit	79/86	1 : 4,000	Over night	Cell Signaling	12640
HA-tag	C29F4	Rabbit	n.a.	1 : 10,000	Over night	Cell Signaling	3724
RBPSUH (RBPJ)	D10A4	Rabbit	61	1 : 2,000	Over night	Cell Signaling	5313
Cleaved Notch-1 (NICD)	D3B8	Rabbit	110	1 : 1,000	Over night	Cell Signaling	4147
Notch1 (NTM/FL)	D1E11	Rabbit	120/300	1 : 1,000	Over night	Cell Signaling	3608
Notch2 (NTM/FL)	D76A6	Rabbit	110/300	1 : 1,000	Over night	Cell Signaling	5732
Notch3 (NTM/FL)	D11B8	Rabbit	90/270	1 : 1,000	Over night	Cell Signaling	5276
Nicastrin	D38F9	Rabbit	110	1 : 1,000	Over night	Cell Signaling	5665
HES1	D6P2U	Rabbit	30	1 : 1,000	Over night	Cell Signaling	11988
Jagged1	28H8	Rabbit	180	1 : 1,000	Over night	Cell Signaling	2620
Jagged2	C23D2	Rabbit	150	1 : 1,000	Over night	Cell Signaling	2210
DLL4	Polyclonal	Rabbit	75-80	1 : 1,000	Over night	Cell Signaling	2589
ADAM9	D64B5	Rabbit	100-115, 75-80	1 : 1,000	Over night	Cell Signaling	4151
TACE	D22H4	Rabbit	135	1 : 1,000	Over night	Cell Signaling	6978
NUMB	C29G11	Rabbit	72/74	1 : 1,000	Over night	Cell Signaling	2756
PEN2	D6G8	Rabbit	13	1 : 1,000	Over night	Cell Signaling	8598
Presenilin 1	D39D1	Rabbit	22	1 : 2,000	Over night	Cell Signaling	5643
Presenilin 2	D30G3	Rabbit	23	1 : 2,000	Over night	Cell Signaling	9979
Anti-rabbit IgG-HRP conjugated	n.a.	Goat	n.a.	1 : 30,000	2 h	Acris	R1364HRP

kD = kilo Dalton, n.a. = not applicable, NICD = Notch intracellular domain, NTM = Notch transmembrane and intracellular domain, FL = full-length, IgG = immunoglobulin G, HRP = horseradish peroxidase

Dual luciferase reporter assay

Tumor cell lines were transfected (X-tremeGENE HP DNA Transfection Reagent, Roche; Amaxa Nucleofector II, Lonza) with Luciferase-reporter vectors (pGL4.14/pGL4.47/pRL, Promega; Cignal™ Pathway Reporter Kit, Qiagen) that either drive the constitutive expression of Renilla-Luciferase for normalization of transfection efficiency, or allow for expression of Firefly-Luciferase driven in the absence or presence of the STAT3 Transcriptional Response Element (Ctrl.-Luc or STAT3-Luc, respectively). Ctrl.-Luc allows for further normalization of STAT3-regulated expression of Firefly-Luciferase. The Renilla-Luciferase vector was co-transfected with either Ctrl.-Luc or STAT3-Luc into STAT3-wild-type or STAT3-mutant variants of LS411N cells, or SW1463 and SW837 cells that had been left untreated or pre-treated with napabucasin, tocilizumab or ruxolitinib (Selleckchem, Munich, Germany), or were genetically modified via RNAi.

Twenty-four hours after transfection, half of the cells were left untreated or stimulated with either 100 ng/ml IL-6 (LS411N and SW837) or 50 ng/ml IL-6 (SW1463) overnight, or with 20 ng/ml Hy-IL-6 for 16 hours overnight. Renilla and Firefly luciferase activities were measured using a microplate reader (Mithras LB940; Berthold Technologies, Bad Wildbad, Germany). Based on the Renilla-normalized values, the Hyper-IL-6-induced STAT3 activity of otherwise untreated cells was calculated as ratio of STAT3-Luc to Ctrl.-Luc ("Ratio STAT3-Luc/Ctrl.-Luc"). The specific STAT3 transcriptional reporter activities of siRNA-treated cells or cells treated with various inhibitors were calculated by further normalizing to Ctrl.-Luc values of untreated and treated cells resulting in the ratio termed "normalized STAT3 activity".

Electrophoretic mobility shift assay

SW837 cells left untreated or stimulated for 30 min with Hy-IL-6 (20 ng/ml) and were lysed in cytoplasmic extraction buffer. Nuclei were isolated by centrifugation and incubated in 50 µl nuclear extraction buffer. After centrifugation at 16,000 g for 15 min and 4°C, the nuclear extracts were mixed with the same amount of cytoplasmic extracts from the same cells. As positive control for GAS binding, lysates of unstimulated or IFN-γ-(50 ng/ml, Biomol, Hamburg, Germany) stimulated HeLa cells were used. For testing of STAT3 binding to the GAS-like element in the RBPJ promotor region, we used [33P]-labeled duplex oligonucleotide probes with 5 bp T overhangs at their 5' end. The sequences of the native and mutated RBPJ fragment and M67 are provided in Supplementary Table S4. The [33P]-labeled duplex oligonucleotides were generated by an end-filling reaction catalyzed by the Klenow fragment (New England Biolabs, Frankfurt am Main, Germany). Four µl of cellular extracts were incubated with 8 µl of EMSA reaction buffer containing 1 ng of the [33P]-labeled probes. For competition experiments, a 750-fold molar excess of unlabeled native RBPJ was added to the reaction and incubated for 15 min at room temperature. Following electrophoretic separation on 8% acrylamide:bisacrylamide gels (29:1), DNA-binding complexes were autoradiographically detected on vacuum-dried gels using the laser phosphorimaging system Typhoon FLA 9500 (GE Healthcare).

Screening STAT3 target genes by RNA-Seq and Opposite Direction Analysis

STAT3 expression was silenced with siRNAs purchased from two different companies (Qiagen or Dharmacon) in separate approaches and using scrambled siRNA as negative control (siCtrl.), with three independent biological replicates either without further treatment or incubation with 20 ng/ml Hy-IL-6 for 16 hours. The sequencing of total RNA samples was conducted at the NGS-Integrative Genomics Core Unit (NIG), University Medical Center Goettingen. Quality and integrity of RNA was assessed with the Fragment Analyzer from Advanced Analytical by using the standard sensitivity RNA Analysis Kit (DNF-471). All samples selected for sequencing exhibited an RNA integrity number > 8. RNA sequencing (RNA-Seq) libraries were generated using 500 ng mRNA of a non-stranded RNA, massively parallel mRNA sequencing approach from Illumina (TruSeq RNA Library Preparation Kit v2, Set A; 48 samples, 12 indexes, Cat. N°RS-122-2001). For accurate quantitation of cDNA libraries, prepared on automation (Beckman Coulter's Biomek FXP workstation), a fluorometric based system, the QuantiFluor™ dsDNA System from Promega was used. The size of final cDNA libraries was determined by using the dsDNA 905 Reagent Kit (Fragment Analyzer from Advanced Bioanalytical) exhibiting an average length of 300 bp. Libraries were pooled and sequenced on the Illumina HiSeq 4000 (Illumina, Inc., San Diego, CA, USA; SE; 1 x 50 bp; 30-35 Mio reads/sample). Sequence images were transformed with Illumina software BaseCaller to BCL files, which was demultiplexed to fastq files with bcl2fastq (version v2.17.1.14). RNA-Seq data were analyzed at the Core Facility, Medical Biometry and Statistical Bioinformatics, Department of Medical Statistics, University Medical Center Goettingen. Quality check was performed using FastQC [13] (version 0.11.5, Babraham Bioinformatics). Reads were aligned to the human reference genome (assembly GRCh38) using STAR [14] (version 2.5.2b). Multiqc [15] (version v1.6. dev0) was used to facilitate quality control on the input data as well as the alignment statistics. Transcript level quantifications were generated using the ensembl annotation (release 93) and the software RSEM [16] (version 1.2.19) to minimize multi-reads. The resulting quantifications have been translated to gene level abundance estimates using tximport [17] (version 1.12.3) to account for sample specific transcript usage and resulting sample specific average transcript lengths. Only fragments with at least three CPMs in at least three samples were retained for the differential expression and downstream analyses. edgeR [18] (version 3.26.6) was used to model gene expression with transfection kit and the experimental conditions: stimulation, knockdown, combined treatment (stimulation and knockdown) as factors. Analyses comparing treatment levels or RNAi-

mediated silencing vs. control were performed as contrast tests. The results were summarized in gene tables with effect size and significance annotation. Resulting *P*-values were adjusted for multiple testing using Benjamini-Hochberg (BH) to control for the false discovery rate (FDR). The number of differentially up- and down-regulated genes was calculated and depicted as volcano plots. Functional enrichment in GO biological processes was analyzed using clusterProfiler [19] (version 3.12.0). For GO biological processes an overrepresentation analysis (ORA) was performed. The results were reported in gene set tables with effect size and significance annotation. All analyses were performed in R [20] (version 3.4.0). Differentially expressed genes were identified for three conditions (siCtrl. vs. siCtrl. + Hy-IL-6; siCtrl. vs. siSTAT3; siCtrl. + Hy-IL-6 vs. siSTAT3 + Hy-IL-6) according to the FDR cut-off of 0.01. Venn diagram analysis was generated using the web-based tool http://bioinformatics.psb.ugent.be/webtools/Venn/. Opposite Direction analysis (ODA) identified genes that were significantly upregulated (FDR cut-off 0.01) upon Hy-IL-6 stimulation of cells and, inversely, downregulated upon STAT3 silencing. A heatmap of ODA genes was generated using Morpheus (https://software.broadinstitute.org/morpheus). The sequencing data and abundance measurement files have been submitted to the NCBI Gene Expression Omnibus (GEO) under the accession number GSE139455. Semi quantitative RT-PCR analysis on total RNA samples was performed using the SensiFAST™ SYBR® No-ROX One-step Kit (Bioline, Memphis, TN) on a BIO-RAD CFX384™ Real-Time PCR Detection System (BIO-RAD, Hercules, CA) as described [11]. The medians of the resulting cycle threshold (Ct) values were normalized to the housekeeping gene *HPRT1* and relative gene expression changes were calculated according the $2^{-\Delta\Delta CT}$ algorithm [21].

Patients, Gene Expression Profiling and Survival Analysis

Pretherapeutic biopsies were obtained from 207 patients with locally advanced rectal cancer. All patients were treated with preoperative chemoradiotherapy, either within or according to the CAO/ARO/AIO-94 and $^{-4}$ trials [22,23]. This project was conducted by the Clinical Research Unit 179 (KFO179), approved by the Ethics Committee of the University Medical Center Goettingen, and with informed consent obtained from all patients. The patients' characteristics were exported from our internal database (SecuTrial, iAS, Berlin, Germany), and are displayed in Supplementary Table S5.

Similarly, the expression levels of *NOTCH1-4* were extracted and listed in Supplementary Table S6. For correlation of gene expression data with clinical parameters, Kaplan-Meier curves displaying disease-free survival (DFS) were generated. Patients were grouped according to gene expression levels above or below the median expression of a particular mRNA. Survival rates were determined using the R package survival, computed by means of the Kaplan-Meier analysis and tested using the Cox proportional hazards model. Disease-free survival was defined as the time from surgery until detection of locoregional or distant recurrence.

Mice

Animal experiments were approved by the German Animal Welfare Act (reference number: 33.9-42502^{-4}-17/2383). Athymic nude Naval Medical Research Institute (NMRI) Foxn1nu/Foxn1nu mice were obtained from Janvier (Janvier-Labs, Le Genest-Saint-Isle, France), respectively. For xenograft transplantation, 2x106 SW1463 cells were subcutaneously injected into the right flank. Experiments started when the tumor reached a volume of about 150 mm^3. For CRT/napabucasin treatment, mice were randomly separated into four different groups: DMSO (n=15), napabucasin (n=15), DMSO + CRT (n=14), and napabucasin + CRT (n=13). The treatment protocol recapitulated clinical conditions described in [11], i.e., fractionated doses of chemotherapy and irradiation, and included intraperitoneal injections of 5-FU (50 mg/kg) and oral application of either DMSO or napabucasin (5 mg/kg), one hour before irradiation with 1.8 Gy (Gulmay,

70 kV, 25 mA and with 0.5 mm Al filtration) for 14 days (total dose of 25.2 Gy). Body weight and tumor volume (volume = (width^2 x length) / 2) were measured thrice weekly. According to the legal termination criterion, mice were sacrificed after tumor volume reached approximately 1,500 mm^3.

References for Supplementary Information

1. Decker, T.; Kovarik, P.; Meinke, A. GAS elements: a few nucleotides with a major impact on cytokine-induced gene expression. *J Interferon Cytokine Res* **1997**, *17*, 121-134, doi:10.1089/jir.1997.17.121.
2. Garbers, C.; Aparicio-Siegmund, S.; Rose-John, S. The IL-6/gp130/STAT3 signaling axis: recent advances towards specific inhibition. *Curr Opin Immunol* **2015**, *34*, 75-82, doi:10.1016/j.coi.2015.02.008.
3. Huynh, J.; Chand, A.; Gough, D.; Ernst, M. Therapeutically exploiting STAT3 activity in cancer - using tissue repair as a road map. *Nat Rev Cancer* **2019**, *19*, 82-96, doi:10.1038/s41568-018-0090-8.
4. Johnson, D.E.; O'Keefe, R.A.; Grandis, J.R. Targeting the IL-6/JAK/STAT3 signalling axis in cancer. *Nat Rev Clin Oncol* **2018**, *15*, 234-248, doi:10.1038/nrclinonc.2018.8.
5. Jones, S.A.; Jenkins, B.J. Recent insights into targeting the IL-6 cytokine family in inflammatory diseases and cancer. *Nat Rev Immunol* **2018**, *18*, 773-789, doi:10.1038/s41577-018-0066-7.
6. Kang, S.; Narazaki, M.; Metwally, H.; Kishimoto, T. Historical overview of the interleukin-6 family cytokine. *J Exp Med* **2020**, *217*, doi:10.1084/jem.20190347.
7. Yu, H.; Lee, H.; Herrmann, A.; Buettner, R.; Jove, R. Revisiting STAT3 signalling in cancer: new and unexpected biological functions. *Nat Rev Cancer* **2014**, *14*, 736-746, doi:10.1038/nrc3818.
8. Fischer, M.; Goldschmitt, J.; Peschel, C.; Brakenhoff, J.P.; Kallen, K.J.; Wollmer, A.; Grotzinger, J.; Rose-John, S. I. A bioactive designer cytokine for human hematopoietic progenitor cell expansion. *Nat Biotechnol* **1997**, *15*, 142-145, doi:10.1038/nbt0297-142.
9. Kopan, R.; Ilagan, M.X. The canonical Notch signaling pathway: unfolding the activation mechanism. *Cell* **2009**, *137*, 216-233, doi:10.1016/j.cell.2009.03.045.
10. Ntziachristos, P.; Lim, J.S.; Sage, J.; Aifantis, I. From fly wings to targeted cancer therapies: a centennial for notch signaling. *Cancer Cell* **2014**, *25*, 318-334, doi:10.1016/j.ccr.2014.02.018.
11. Spitzner, M.; Roesler, B.; Bielfeld, C.; Emons, G.; Gaedcke, J.; Wolff, H.A.; Rave-Frank, M.; Kramer, F.; Beissbarth, T.; Kitz, J., et al. STAT3 inhibition sensitizes colorectal cancer to chemoradiotherapy in vitro and in vivo. *Int J Cancer* **2014**, *134*, 997-1007, doi:10.1002/ijc.28429.
12. Grade, M.; Hummon, A.B.; Camps, J.; Emons, G.; Spitzner, M.; Gaedcke, J.; Hoermann, P.; Ebner, R.; Becker, H.; Difilippantonio, M.J., et al. A genomic strategy for the functional validation of colorectal cancer genes identifies potential therapeutic targets. *Int J Cancer* **2011**, *128*, 1069-1079, doi:10.1002/ijc.25453.
13. Andrews, S. FastQC A Quality Control Tool for High Throughput Sequence Data. Availabe online: https://github.com/s-andrews/FastQC (accessed on

14. Dobin, A.; Davis, C.A.; Schlesinger, F.; Drenkow, J.; Zaleski, C.; Jha, S.; Batut, P.; Chaisson, M.; Gingeras, T.R. STAR: ultrafast universal RNA-seq aligner. *Bioinformatics* **2013**, *29*, 15-21, doi:10.1093/bioinformatics/bts635.

15. Ewels, P.; Magnusson, M.; Lundin, S.; Kaller, M. MultiQC: summarize analysis results for multiple tools and samples in a single report. *Bioinformatics* **2016**, *32*, 3047-3048, doi:10.1093/bioinformatics/btw354.

16. Li, B.; Dewey, C.N. RSEM: accurate transcript quantification from RNA-Seq data with or without a reference genome. *BMC Bioinformatics* **2011**, *12*, 323, doi:10.1186/1471-2105-12-323.

17. Soneson, C.; Love, M.I.; Robinson, M.D. Differential analyses for RNA-seq: transcript-level estimates improve gene-level inferences. *F1000Res* **2015**, *4*, 1521, doi:10.12688/f1000research.7563.2.

18. Robinson, M.D.; McCarthy, D.J.; Smyth, G.K. edgeR: a Bioconductor package for differential expression analysis of digital gene expression data. *Bioinformatics* **2010**, *26*, 139-140, doi:10.1093/bioinformatics/btp616.

19. Yu, G.; Wang, L.G.; Han, Y.; He, Q.Y. clusterProfiler: an R package for comparing biological themes among gene clusters. *OMICS* **2012**, *16*, 284-287, doi:10.1089/omi.2011.0118.

20. Team, R.C. R: A Language and Environment for Statistical Computing. Availabe online: https://www.R-project.org/ (accessed on

21. Livak, K.J.; Schmittgen, T.D. Analysis of relative gene expression data using real-time quantitative PCR and the 2(-Delta Delta C(T)) Method. *Methods* **2001**, *25*, 402-408, doi:10.1006/meth.2001.1262.

22. Sauer, R.; Becker, H.; Hohenberger, W.; Rodel, C.; Wittekind, C.; Fietkau, R.; Martus, P.; Tschmelitsch, J.; Hager, E.; Hess, C.F., et al. Preoperative versus postoperative chemoradiotherapy for rectal cancer. *N Engl J Med* **2004**, *351*, 1731-1740, doi:10.1056/NEJMoa040694.

23. Rodel, C.; Liersch, T.; Becker, H.; Fietkau, R.; Hohenberger, W.; Hothorn, T.; Graeven, U.; Arnold, D.; Lang-Welzenbach, M.; Raab, H.R., et al. Preoperative chemoradiotherapy and postoperative chemotherapy with fluorouracil and oxaliplatin versus fluorouracil alone in locally advanced rectal cancer: initial results of the German CAO/ARO/AIO-[4] randomised phase 3 trial. *Lancet Oncol* **2012**, *13*, 679-687, doi:10.1016/S1470-2045(12)70187-0.
